# Supplementary material for: Host heterogeneity and epistasis explain punctuated evolution of SARS-CoV-2
Source: PLoS Comput Biol. 2023 Feb 15;19(2):e1010896. doi: 10.1371/journal.pcbi.1010896 (PMC9974118; doi:10.1371/journal.pcbi.1010896)
Supplement: S1 Appendix — (PDF) [file pcbi.1010896.s009.pdf]

Diversity dynamics based on US sequences

In the main text, we based our analysis on sequence data from the United Kingdom, since the UK genomic surveillance was extensive. However, the overall results remain the same if one instead considers United States sequence data. The exact timing of the variant transitions differ – most notably, the interval between the Alpha and Delta transitions is shorter – but the qualitative features are similar. Variant transitions continue to be associated with large jumps in Hamming distance, indicative of saltation. The temporal Hamming distribution for the US is shown in Fig A. Due to the shorter interval, the Alpha and Delta transitions are not as clearly separated as in the UK data, and the Alpha transition appears somewhat drawn out. As demonstrated in S6 Fig, this could be a result of the presence of greater spatial effects.

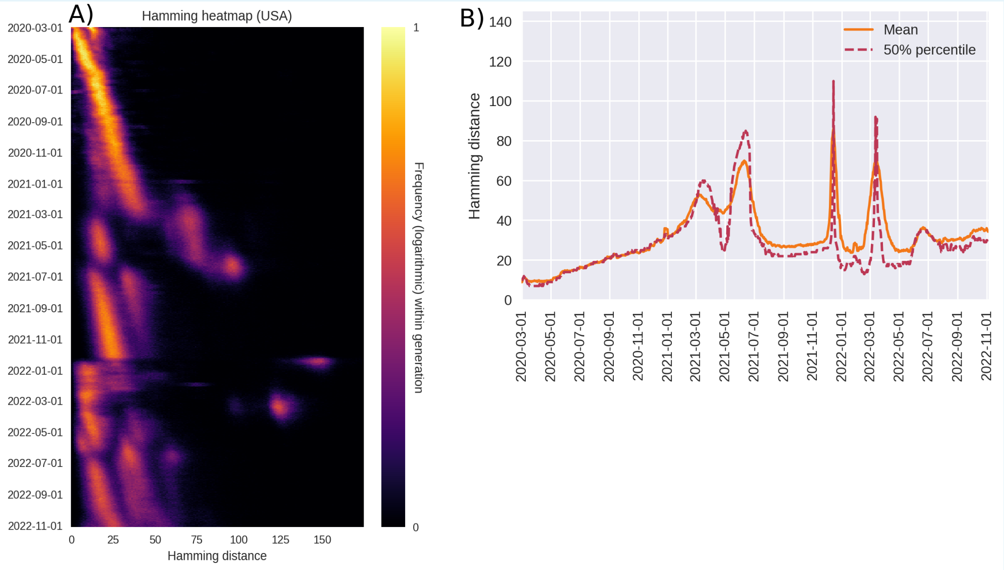

S1 Appendix Fig A. Hamming distance analysis analogous to that of Fig 1 of the main text, except based on United States sequence data rather than British sequences. **A)** The full, time-dependent Hamming distribution (US data, GenBank via Nextstrain). **B)** Time evolution of the mean and median pairwise Hamming distances.
