# Supplementary material for: Host heterogeneity and epistasis explain punctuated evolution of SARS-CoV-2
Source: PLoS Comput Biol. 2023 Feb 15;19(2):e1010896. doi: 10.1371/journal.pcbi.1010896 (PMC9974118; doi:10.1371/journal.pcbi.1010896)
Supplement: S2 Appendix — (PDF) [file pcbi.1010896.s010.pdf]

Fitting the origin-centered Hamming distribution

Our analysis is mainly centered around the dynamics of diversity, as measured by the dissimilarity of SARS-CoV-2 genomes which are in circulation at a given point in time (Fig 1 of the main text). Our primary goal was to formulate a dynamical model which could qualitatively replicate this pattern as parsimoniously as possible.

In this section, we additionally consider the distance between circulating genomes and the *origin* (meaning the sequence Wuhan-Hu-1, GenBank reference sequence accession number MN908947.3). In Fig A, we show the distribution of distances between circulating genomes and this reference sequence, which we will call the *absolute* Hamming distance. The data analysis workflow in creating this plot is analogous to that detailed in S1 Fig, but instead of continuously picking *pairs* of genomes, we pick single genomes which are then compared with the reference genome. This also means that, given N genomes within a given time window, there can be only N data points with this method. This is in contrast to the pairwise comparison in Fig 1 of the main text, where N genomes give rise to $N(N-1) \sim N^2$ possible pairings.

In Fig A, we see that the absolute Hamming distance at first increases approximately linearly with time until the Alpha transition, at which point a large jump in the absolute Hamming distance is observed. Interestingly, the transition from Alpha to Delta is associated with a slight decrease in this distance. The Omicron transition is again associated with a large increase in the absolute Hamming distance.

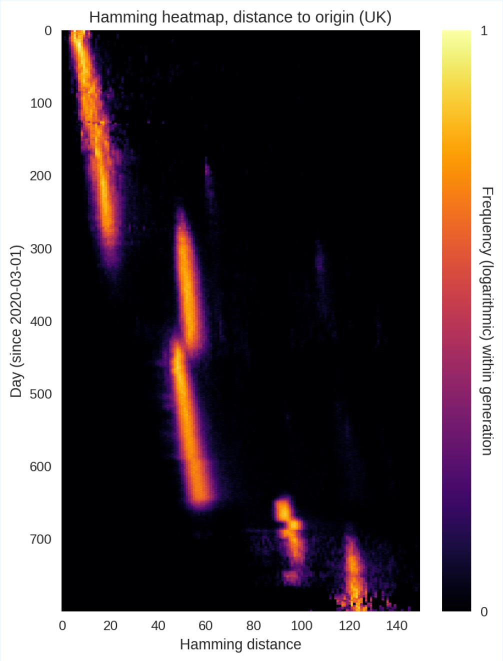

S2 Appendix Fig A. Time evolution of the *absolute* Hamming distance, i.e. the distance to the origin (defined as the Wuhan-Hu-1 reference sequence). UK sequence data.

One possible explanation for such a decrease in absolute Hamming distance is that prolonged infections with an earlier variant have occurred in some individuals or a population, albeit without accompanying accelerated evolution. Once the unobserved lineage spills into

the sampled population, it may lead to a variant transition despite being closer to the ancestral variant than to the currently dominating one.

In terms of our model, this kind of dynamics can be quite simply incorporated. While it is not impossible to see decreases in absolute Hamming distance in the simple model formulation behind Fig 3 of the main text, it is highly statistically unlikely, since each saltation happens on the basis of the genomes present in the previous generation.

The very simplest way to incorporate the possibility of decreasing absolute Hamming distance is to assume that a certain fraction of the population are initially infected with the ancestral strain, but that their infections are prolonged (and that they thus only transmit the disease much later). If the pathogen undergoes mutation within these hosts, it is possible to obtain a pattern such as the one shown in Fig B, panel B. As evidenced by panel A of that same figure, the pairwise 'relative' Hamming distance between genomes in the same generation is not qualitatively affected by this addition to the model. Transitions are still driven by large saltations, and the (relative) Hamming distance is characterized by periods of linear growth punctuated by large increases and subsequent collapses in diversity. The technical details of this addition to the model can be found in the Materials and Methods section.

While allowing for persistent infections with the initial variant is of course a very simple variation on the base model, it does show that prolonged infections with previous variants can account for occasional sudden decreases in the absolute Hamming distance.

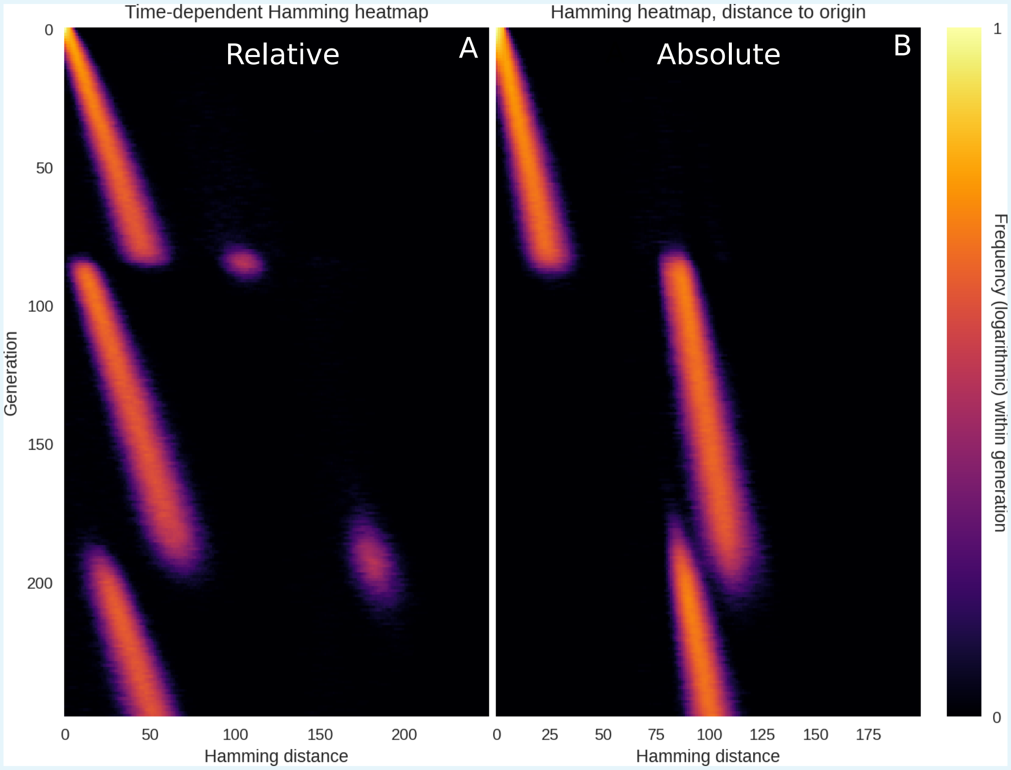

S2 Appendix Fig B. Simulation with persistent infections with original variant. When the original variant persists in a small fraction of the population, absolute Hamming distance can decrease at variant transitions. **A)** The 'relative' Hamming distance, the same quantity that was plotted in e.g. main text Figs 1 and 3. It measures the dissimilarity between concurrently circulating pathogen genomes. **B)** The 'absolute' Hamming distribution, measuring the distance between circulating pathogen genomes and the reference sequence, namely that of the initial variant.
