# Supplementary material for: Host heterogeneity and epistasis explain punctuated evolution of SARS-CoV-2
Source: PLoS Comput Biol. 2023 Feb 15;19(2):e1010896. doi: 10.1371/journal.pcbi.1010896 (PMC9974118; doi:10.1371/journal.pcbi.1010896)
Supplement: S3 Appendix — (PDF) [file pcbi.1010896.s011.pdf]

Variant dynamics under strain-specific immunity

During all of 2022, different Omicron subtypes have dominated in terms of prevalence. As shown in the main text, the transitions to BA.1 and BA.2 were both marked by saltational events. However, in the period since BA.2 became dominant, the picture of long stretches of linearly increasing Hamming distance punctuated by large saltations has not been apparent. Instead, the prevailing picture has been one of rapid variant turnover and coexistence of multiple variants.

While an exhaustive treatment of this phenomenon is beyond the scope of the current manuscript, we wish to sketch the outlines of an explanation, in terms of the model and observations introduced in the paper. As noted in the main text, the first major transition, ancestral→Alpha, happened in a background with low population immunity – no vaccines and very limited infection-induced ("natural") immunity. Thus, the fitness advantage was due to an increase in inherent transmissibility. With the transitions from Alpha to Delta and Delta to Omicron (BA.1), a high level of vaccine coverage had been attained in e.g. the UK, owing to vaccines based on the ancestral variant.

However, we are presently in a situation where the immunity landscape seen by new viral strains is highly heterogeneous. Most people in highly-sequenced countries are vaccinated against the ancestral strain, some have received omicron-based booster shots and many have been infected with one variant or another at varying time points. This means that a prospective variant does not merely see a homogeneous pool of susceptibles. And with immunity having a strain-specific component [1, 2, 3], different variants do not see the *same* pool of susceptibles.

For this reason, a new variant may thrive not because of any inherent advantage in terms of transmissibility, but simply because it is antigenically different enough to a previous strain to (re)infect potions of the population. And variants may coexist because they can, to some extent, subsist on different 'resources' (different parts of the population being susceptible to different new variants due to heterogeneity in immunity).

In order to probe the consequences of such a scenario, we implement (tunably) strain-specific immunity. In an agent-based SIRS implementation of the model, we add strain-specific immune memory by equipping each agent i with an $N_e \times 2^{L_e}$ matrix Imm_i taking on real values between 0 and 1.
If the matrix element $\mathrm{Imm}_i(n, m)$ takes on the value 1, the i'th agent has maximal immune recognition of the m'th configuration of the n'th epitope. If the value is 0, the agent has no immune memory of that particular configuration.
The waning immunity of the SIRS model is then implemented by letting the immunity matrix Imm decay at a rate ω. In other words, in each time step, we let

$$\mathrm{Imm} \rightarrow (1 - \omega \mathrm{d}t)\mathrm{Imm}$$

When the i'th agent is infected with a particular variant, the corresponding elements of Imm_i are set to 1. That is the strain-specific part. However, a level of strain-transcending immunity can also be be implemented by setting the remaining elements of Imm_i to a value $\xi \in [0, 1]$ upon infection. If e.g. $\xi = 0.5$, newly recovered individuals will experience a broad immunity at half the strength of their strain-specific immunity.

Below, we run simulations of this type with rate of immunity waning $\omega = 1/25$ (measured in units of 1/(generation time)). To look separately at the effects of strain-specific immunity, we let $\delta R_H = 0$, meaning that no inherently more transmissible variants can arise. The remaining parameters are total population size $N = 2 \times 10^6$, reproductive number in a naive

population $R_0 = 1.4$, $\delta R_L = -\infty$, frequency of saltation $\varepsilon = 10^{-4}$.
We run simulations with different levels of cross-immunity, $\xi = 0$, $\xi = 0.5$, $\xi = 1.0$.

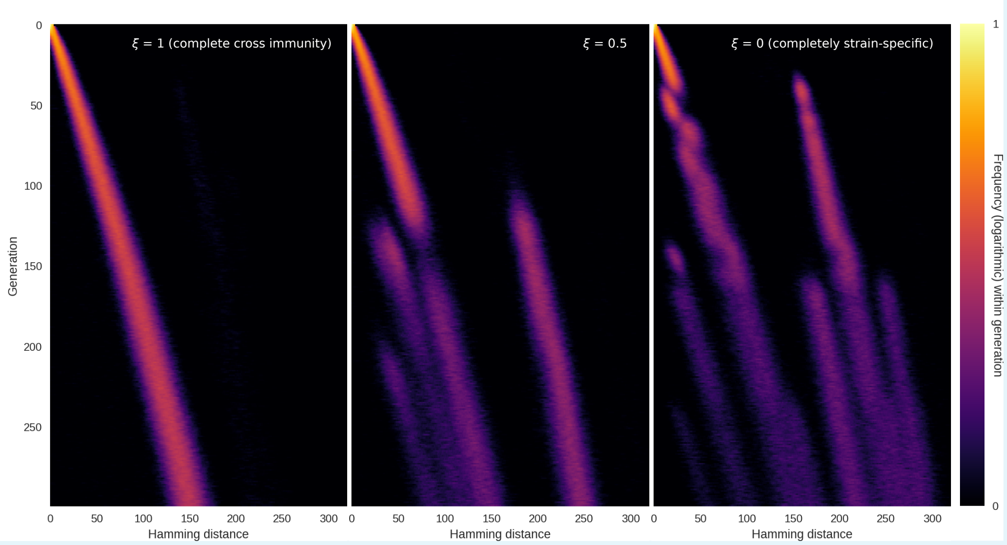

S3 Appendix Fig A. Hamming dynamics under partially strain-specific immunity. In these simulations, we assume that no inherent fitness advantages (i.e. increases in R_0) are evolutionarily accessible. Instead, all fitness advantages are due to evasion of (partially) strain-specific immunity. **Left:** Here, we let the cross immunity parameter $\xi = 1$, so that immunity is completely strain-transcending. **Middle:** Here, $\xi = 0.5$, meaning that immunity is partially strain-specific. **Right:** We let $\xi = 0$, corresponding to completely strain-specific immunity, i.e. without any cross immunity.

What Fig A shows, conceptually, is that:

- Partially strain-specific immunity can exert an evolutionary pressure towards novelty, driving the occurrence of new viral strains.
- Several strains may co-circulate under the influence of the heterogeneous immunity landscape resulting from previous infection with multiple different variants.

While by no means an exhaustive description of the late-2022 situation of SARS-CoV-2 co-circulation and rapid variant turnover – which we deem outside the scope of this paper – these simulations do provide the building blocks for such an analysis.

In Fig B, we run simulations similar to those of Fig A, except that we introduce a new, intrinsically more transmissible variant in a few individuals at $t = 250$. The effects on the Hamming dynamics turns out to depend heavily on the degree of cross immunity between strains. When there is partial cross immunity ($\xi = 0.5$), the introduction of a fit strain collapses the Hamming distribution, leading to a unimodal distribution (Fig B, right). However, if immunity is completely strain-specific, the addition of a highly transmissible strain is not accompanied by a collapse in diversity. In the former case, the different variants to a large extent ‘see’ the same susceptibles and thus subsist on the same ‘resource’, while they can co-circulate more or less independently in the $\xi \to 0$ limit. These simulation highlight how the plurality of possible future developments of the SARS-CoV-2 pandemic are, to a high degree, modulated by the nature of strain-transcending immunity and evasion thereof.

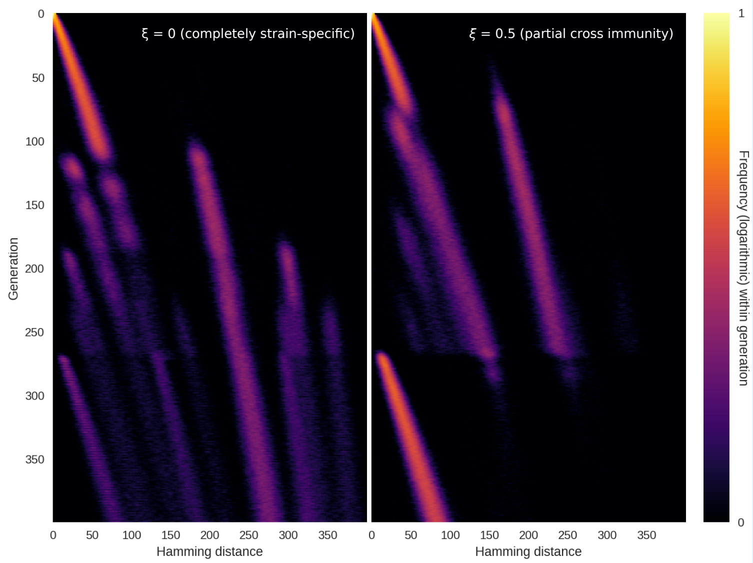

S3 Appendix Fig B. Highly transmissible variant emerging in heterogeneous immunity landscape. In these simulations, we explore what happens when an intrinsically more transmissible variant emerges in a scenario with several co-circulating variants in a heterogeneous immunity landscape. **Left:** At $\xi = 0$, there is no cross-immunity (e.g. immunity is completely strain-specific). In this case, co-circulation continues although the more fit variant is introduced at time $t = 250$. The new variant shows up as a peak at low Hamming distance, becoming visible around $t = 175$. **Right:** At $\xi = 0.5$, there is appreciable cross-immunity. In this case, the emergence of a new, more transmissible variant homogenizes the genomic landscape, with a single peak at low Hamming distance beginning to dominate around $t = 175$.

References

1. Bates, T. A., McBride, S. K., Winders, B., Schoen, D., Trautmann, L., Curlin, M. E., and Tafesse, F. G. (2022). Antibody response and variant cross-neutralization after sars-cov-2 breakthrough infection. *Jama*, 327(2):179–181.

2. Ju, B., Fan, Q., Wang, M., Liao, X., Guo, H., Wang, H., Ge, X., Liu, L., and Zhang, Z. (2022). Antigenic sin of wild-type sars-cov-2 vaccine shapes poor cross-neutralization of ba. 4/5/2.75 subvariants in ba. 2 breakthrough infections. *Nature Communications*, 13(1):1–6.

3. Laurie, M. T., Liu, J., Sunshine, S., Peng, J., Black, D., Mitchell, A. M., Mann, S. A., Pilarowski, G., Zorn, K. C., Rubio, L., et al. (2022). Sars-cov-2 variant exposures elicit antibody responses with differential cross-neutralization of established and emerging strains including delta and omicron. *The Journal of infectious diseases*, 225(11):1909–1914.
