# Supplementary material for: Host heterogeneity and epistasis explain punctuated evolution of SARS-CoV-2
Source: PLoS Comput Biol. 2023 Feb 15;19(2):e1010896. doi: 10.1371/journal.pcbi.1010896 (PMC9974118; doi:10.1371/journal.pcbi.1010896)

Response to reviewers – “Host heterogeneity and epistasis explain punctuated evolution of SARS-CoV-2”

0) Response to both reviewers

First, we would like to thank both reviewers for their useful and insightful comments.

Before we address the individual points, we would like to address an interesting and fundamental question which was raised by both referees (in slightly different forms).

The question essentially boils down to the following:
“What if the pathogen spreads in an isolated/unsequenced population, and a new variant arises *without* saltation in this population (i.e. without multi-mutation leaps). Can this give rise to a saltation-like signature once the new variant spills into the sequenced population?”

In short, we show below that the answer is negative: this scenario will not result in a saltation-like pattern.

To probe this question, we performed a simulation with two subpopulations which are initially isolated from one another. We think of Subpopulation 2 as the one from which we have access to sequencing data, and Subpopulation 1 as the one in which a new variant emerges.
At a later stage, we put the two populations into contact with one another and keep track of the effect that the resulting spill-over has on the Hamming distribution.

The scenario is simulated in the following way:

- Let the model population consist of two subpopulations, Subpopulation 1 and Subpopulation 2. Initially, the same variant spreads among both populations.
- At first - until t=75 generations in our simulation - the two populations are completely isolated from one another (inter-population transmission rate 0)
- At t=75, the two populations are put into contact with each other (inter-population transmission rate 0.5, meaning that each contact made is as likely be with someone from the other population as with someone from one’s own population)
- If at some time before t=75, a new, more transmissible variant arises in one subpopulation (Subpopulation 1 in the example below), this variant will spread to the other subpopulation after t=75 and come to dominate both subpopulations.

Such a simulation pans out in the following way:

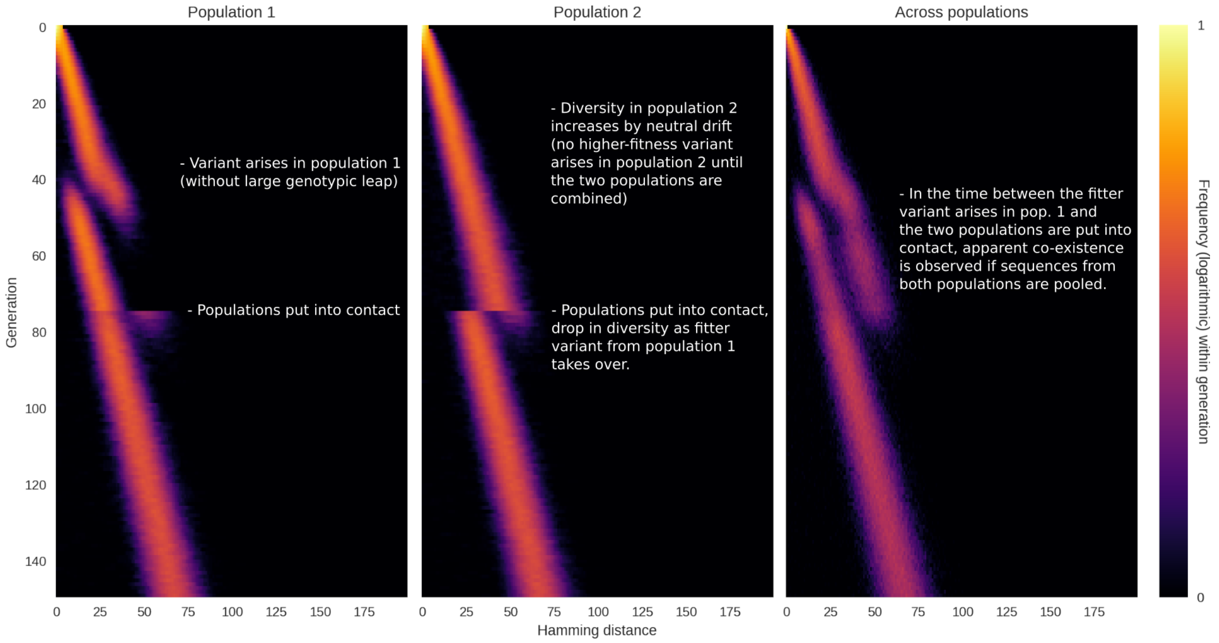

A new variant arises by only a few single-point mutations in population 1 (leftmost panel) around *t*=30 and comes to dominate that population around *t*=40.
At *t*=75, the two populations come into contact, and the fitter variant spreads to population 2 as well (middle panel). Here, it gives rise to a rapid drop in diversity without any saltation-like signature.
The rightmost panel shows the Hamming distribution that would arise if sequences were compared across subpopulations. In this case, variant coexistence is observed from t=40 to t=75, after which a single variant dominates.

The upshot is thus that a variant arising in population 1 and later spilling into population 2 will not – from the perspective of population 2 – appear as a saltation.
Our intuition behind this is as follows:
In the absence of saltation (in the sense of multi-mutation leaps), any two linear transmission chains will diverge from each other at the same rate, genomically speaking. This holds whether or not the two lineages are geographically separated and is determined by the 'molecular clock'.
That this is so is also borne out in the observations, since the typical Hamming distances are similar whether we pool all sequenced within the UK, the US or the whole world (we have added a global Hamming distribution to supplementary figure S7).
We do agree, though, that it is a remarkable fact that sequences within e.g. Denmark are almost as different from one another as sequences from different countries. We checked this, as Denmark is a good example of a high-sequencing but geographically small country. (This does of course not hold exactly during transitions, where the transition may have occurred in one country but not in another, but during the long periods of genetic drift in between transitions, it does).

We have included these simulations and the corresponding discussion in the section on *Epidemic dynamics and spatial structure* near the end of the Results section of the manuscript.

1) Response to reviewer #1

Summary: This paper analyzed SARS-CoV-2 sequence data using Hamming distance to find good evidence for evolution of this virus primarily through saltation with epistasis. They validate their finding using simulations. Overall, I am impressed with the quality of this paper. It is a very useful contribution to the understanding the evolution of this virus.

Author response: We thank the referee for the kind words! We hope that the methods employed in this paper can be a valuable supplement to existing methods in variant surveillance and phylogenetic mapping.

I have a few questions and concerns listed below. I hope the authors will address these points and incorporate them into the paper.

1. The authors claim that most of the evolution of SARS-CoV-2 happens in immunocompromised individuals in whose immune landscape the virus has greater freedom to evolve. Although this makes sense, I would like to see a discussion of other possible mechanisms that might create the kinds of saltation events observed. These may happen gradually in a much larger fitness landscape than what is captured by databases consisting of sequences from symptomatic individuals. In the discussion section the authors consider "reverse zoonosis" to create a mixed epistatic landscape connecting humans to animals. A possible discussion of other mechanisms that might also create a larger epistatic landscape (list given below) could perhaps also be included.
Author response: Yes, we are very interested in exploring alternate explanations, both in terms of mechanisms behind saltations and the possible effects (or lack thereof) of population dynamics on the Hamming distributions. We will go through the suggestions one by one.
We understand exactly what you refer to when writing of "other possible mechanisms that might create the kinds of saltation events observed. These may happen gradually in a much larger fitness landscape". Refer to Section 0 above for a discussion of whether gradual (but unobserved) evolution could appear as an apparent saltation.

Regarding the point about sequences from (a)symptomatic individuals specifically, see our reply further down.

• Changes in binding location from lung, lower respiratory tract to upper respiratory tract to make the virus more infective and less virulent (there is evidence that this is happening in the variants which have emerged since March-April 2020). This adaptation might create sufficient drift followed by a few mutations for a new variant to appear suddenly and be seen as saltation . Is this a possibility?
Author response: An increase in transmissibility by itself is not likely to give a saltation-like signal in the Hamming distribution, but it *is* likely to increase the frequency at which new variants emerge and thus we might expect to see variants replace each other more frequently (or co-circulate – see below, where we also discuss some additions to the paper which relate to this point!).
Our SIRS simulations (which we have re-done in a full agent-based framework) also underscore this point: although the force of infection varies strongly over time, the Hamming

distribution is largely unaffected. That is, the form of the observed Hamming distribution is similar to what we obtained in our (simpler) constant-prevalence simulations (Fig. 3)

• Effect of asymptomatic infected individuals in whom the virus may evolve without being sequenced and create novel variants. The pool of asymptomatic infected individuals (the "dark matter" of this pandemic) may be very large compared to the identified, symptomatic cases, particularly as the virus becomes more infective and less virulent.
Author response: There are a few things to take into account here. First there is the question of whether (unsequenced) asymptomatic individuals could be the source of apparent saltations. Next there is the suggestion that this could be related to the size of the (unsequenced) asymptomatic 'population'.

First, it should be mentioned that screening programmes such as those implemented in the UK and Denmark are likely to – and indeed did – catch many asymptomatic cases, since testing was frequently done for the purpose of showing a negative test.
Asymptomatic testing has been one of the central pillars of the UK COVID testing programme, and thus sequences will include many samples from asymptomatic COVID-positive individuals, see e.g. this report on gov.uk:
https://www.gov.uk/government/publications/technical-report-on-the-covid-19-pandemic-in-the-uk/chapter-6-testing

The second point that the referee raises, we have covered in Section 0 of our response, i.e. how a spill-over from an "unobserved" (i.e. unsequenced) population shows up in the Hamming distribution.

• Changes induced by vaccination targeted to specific variants may create favorable landscapes for new variants.
Author response: Yes! We completely agree. The same holds for infection-induced ("natural") immunity if it is to some extent strain-specific (i.e. incomplete cross immunity).
In fact, referee 2 asked a related question, having to do with the more recent developments (rapid succession of variants and co-circulation of multiple variants). We have added an appendix to the manuscript, *Appendix 3 – Variant dynamics under strain-specific immunity*, in an attempt to show how these observations can be understood within our model framework.

Note that we have also updated the manuscript with the newest sequence data available to us.

• Changes in behavior of individuals, relaxation of quarantine, mask requirements, increased travel, more time spent in areas of poor vaccination (aircraft) or with larger groups of people as controls are relaxed might also create new pools of infected individuals who might potentially provide new variants.
• Opening of schools, colleges, and businesses.
Author response: We agree that these changes in behaviour could contribute to an increased rate of emergence of new variants, simply because the genotypic landscape would be 'explored' faster by the pathogen (i.e. a 'frequency dependence' of emergence of new variants). However, this frequency dependence would not in and of itself give a

saltation-like signature in the Hamming distribution, although it might mean that the next saltation was reached faster.

2. The authors analyze mostly data from first world countries (UK, USA). However, one would expect much greater evolution of disease in third world or densely populated countries with poor health services, such as Africa, India, the Middle East. Would it be possible to include and analyze at least a few sequences from such countries?

Author response: Unfortunately, the amount of sequences available outside of Europe and North America is very low, so we cannot obtain a similar plot quality. However, if we pool all sequences from outside Europe and North America, we can obtain a plot that is clear enough to show that the typical Hamming distances are similar to the UK and US-based plots:

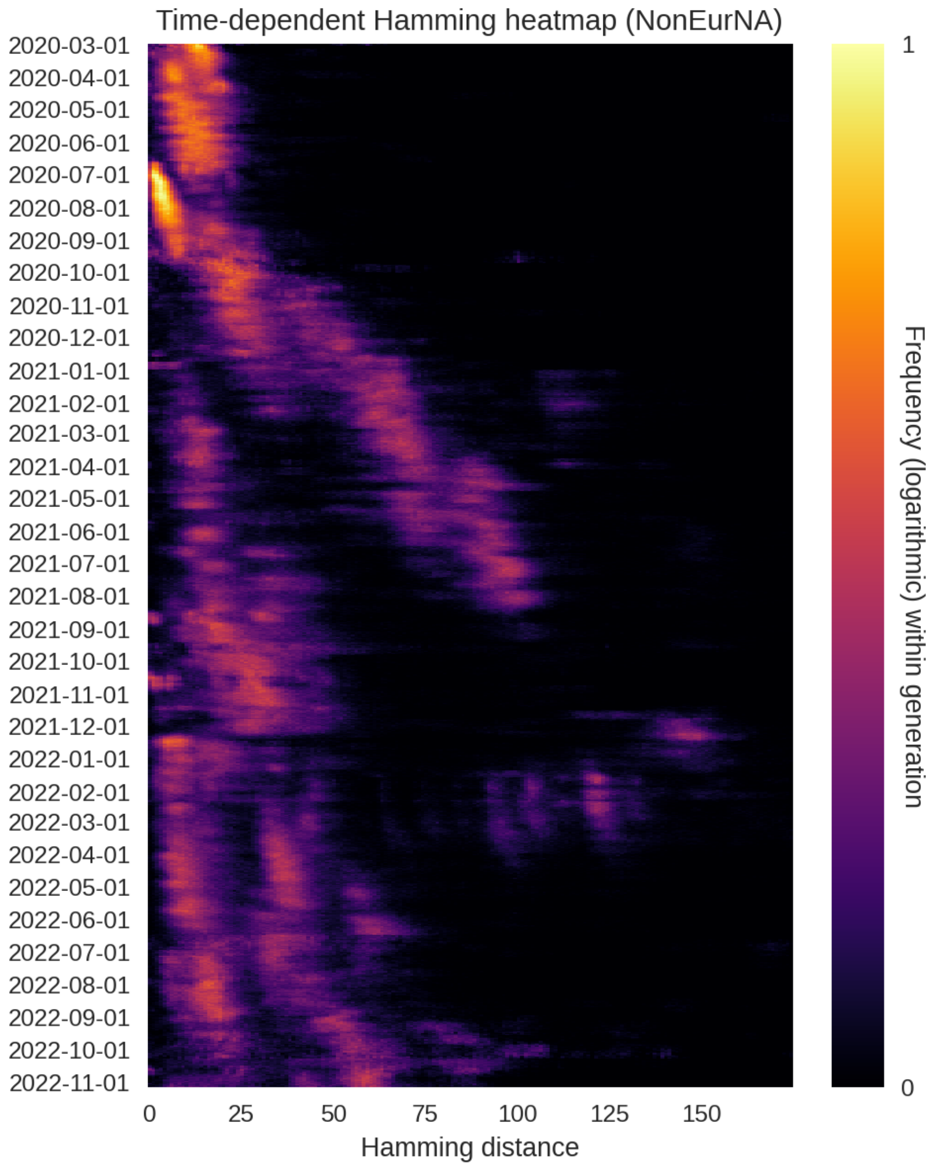

We see that the typical Hamming distances involved are indeed similar to the UK and US-based plots. However, the transitions are much more “smeared out” and a higher degree of coexistence is observed. This is consistent with what we observed regarding spatial

effects in Fig. S6 and what we saw in our simulations of two disconnected populations above.
We have included this analysis along with a Hamming distribution based on all globally available sequences in supplementary figure S7, which we also discuss in the Results section as well as in the Discussion.

3. Is there sequence data from random sampling of the UK population to identify asymptomatic carriers? If so, it would be interesting to check whether these individuals have non-synonymous mutations that might bridge the gap between saltation events.

Author response: As mentioned earlier, asymptomatic testing has been one of the central pillars of the UK COVID testing programme, so the sequences do include many samples from asymptomatic COVID-positive individuals. However, with the metadata available to us, we cannot directly link symptom status to sequences.

4. On page 6, referring to within patient evolution in immunocompromised individuals the authors state that “the most well-documented is perhaps elevated mutation in immunocompromised individuals.” This is an important point and needs a reference.

Author response: We agree - thank you for pointing this out. In fact, further evidence in this direction has emerged since the manuscript was submitted. Notably, a paper by Khatamzas et al in Nature Communications (https://www.nature.com/articles/s41467-022-32772-5) details an immunocompromised patient with a 156-day (ultimately fatal) SARS-CoV-2 infection, who was also treated with convalescent plasma. They document how a slew of mutations arose over the course of the infection and became fixed in the predominant variants sampled from the patient. We have added this reference, along with others which document prolonged infections and the occurrence of multiple mutations in immunocompromised hosts.

This is a well written and timely paper. The point it makes about universal and equitable distribution of vaccines worldwide is an important conclusion.

Author response: Thank you!

2) Response to reviewer #2

The authors provide results of several important types. First, they perform a detailed analysis of genomic data from Covid-19 infections in the UK over an extended period, and calculate the Hamming distance between genomes in the set, determining the genomic diversity over time. They also do this for influenza data, although the data available is not as extensive and high quality. They note that the Covid-19 data appears to have a punctuated evolution, with genetic drift alternating with large increases of diversity followed by a crash. Influenza does not seem to show the same sharp peaks, and the authors seek to model this defining feature of the Covid-19 data. They posit that it is due to genetic saltation, rare but large jumps in critical genetic sequences that enable the virus to traverse deep "valleys" in a fitness landscape. They model this with a commendably simple but effective model, and also add SEIR modeling to include the effects of different populations. In all cases they find they need to have saltational evolution in order to get the qualitative features of the data.

Author response: We would like to thank the referee for the very precise summary.

The paper is very clearly written in understandable colloquial English, the figures are all of them excellent and clear (although not clear without viewing the figure caption, since there is precious little identifying information on each figure. However, since in any publication setting, the figures will appear with their captions, I do not list this as something to be amended.) The Supplements are also commendably short, and yet clearly written and on topics of relevance to the main paper. The supplementary figures are likewise very useful.

Author response: Thank you – we take the point about some figures being a little sparse and have added some more annotations to figures 2, 3, 4, 5 and 6. Now e.g. the Hamming distribution plots of figures 3 and 4 have a bit of text to say which simulation they represent (with or without saltational dynamics/epistasis). In the same vein, the in-figure legend of Figure 5 now explicitly states that we are plotting the final reproductive number relative to the initial one ($R_0^{final}/R_0^{initial}$). We hope these changes have improved the clarity of the figures!

There are only a very few points that need addressing.

1) The word "important" is repeated twice in a row, the only typo I found. This is on page 3, the paragraph two above the Results section. Search for "important important" in the paper and it will be found.

Author response: Thank you! We have now fixed this typographical error.

2) (a) This next point is much more pertinent, and should be addressed in the manuscript. Namely, the time dependence of genetic diversity can suggest another effect, independent of any saltational mutations: the punctuated nature of people's interactions in this pandemic, very different dynamics than happens with influenza. A sudden law to shut down businesses, a sudden law to mandate masks, a closing of schools, then in reverse, the masks off, then back on a few months later, schools open but with masks, then suddenly masks off, then suddenly people gathering, then larger groups, etc.

In other words, could the jumps in genetic diversity be due not to saltational mutation, but rather if everyone's mutation was fairly slow, but people being separated, the mutation was for extended periods in many different directions, then suddenly people appeared out with their new mutations and infected others? Many, many people got Covid who did not necessarily go to the doctor and get sequenced, especially with the later milder versions. It could seem that this "punctuated appearance" of people and groups could also just as easily explain the sudden jumps.
Author response: In Section 0 above, we have investigated the question "What if the disease evolves (slowly) in separated populations, and a new variant arises *without* saltation (i.e. without multi-mutation leaps) in one population?" and whether this can give rise to a saltation-like signature once the new variant spills into the sequenced population.

Superspreader events, for example, especially if some low-immunity individuals attended (say with a higher rate of mutation but not a saltational one).
Author response: While we have modelled each jump as owing to saltational evolution within one individual, we agree that it is possible that the observed jumps could have occurred as a product of accelerated evolution in a chain of a few individuals. The virus would then have to accumulate mutations at a higher average rate within these individuals, resulting in a visible jump in the Hamming distribution. We have added a few sentences to this effect to the Discussion section (along with a supplementary figure showing the Hamming distribution if based only on sequences from *outside* Europe and North America).

(b) In a second related question, how can a presumably constant (low) rate of saltational viral evolution explain the accelerated appearance of closely related new strains of the virus? This is data not covered in the manuscript, more recent, but surely the authors are aware of it. Many, many new variants, not the well-separated peaks of the author's results. But could be explained (?) by more and more people finally coming out without masks, everyone with their own new variant.
Author response: Yes, we have followed the recent developments with great interest! We have extended our Hamming analysis based on the most recent sequence data available to us.
Below are the plots for the UK and the entire world, respectively:

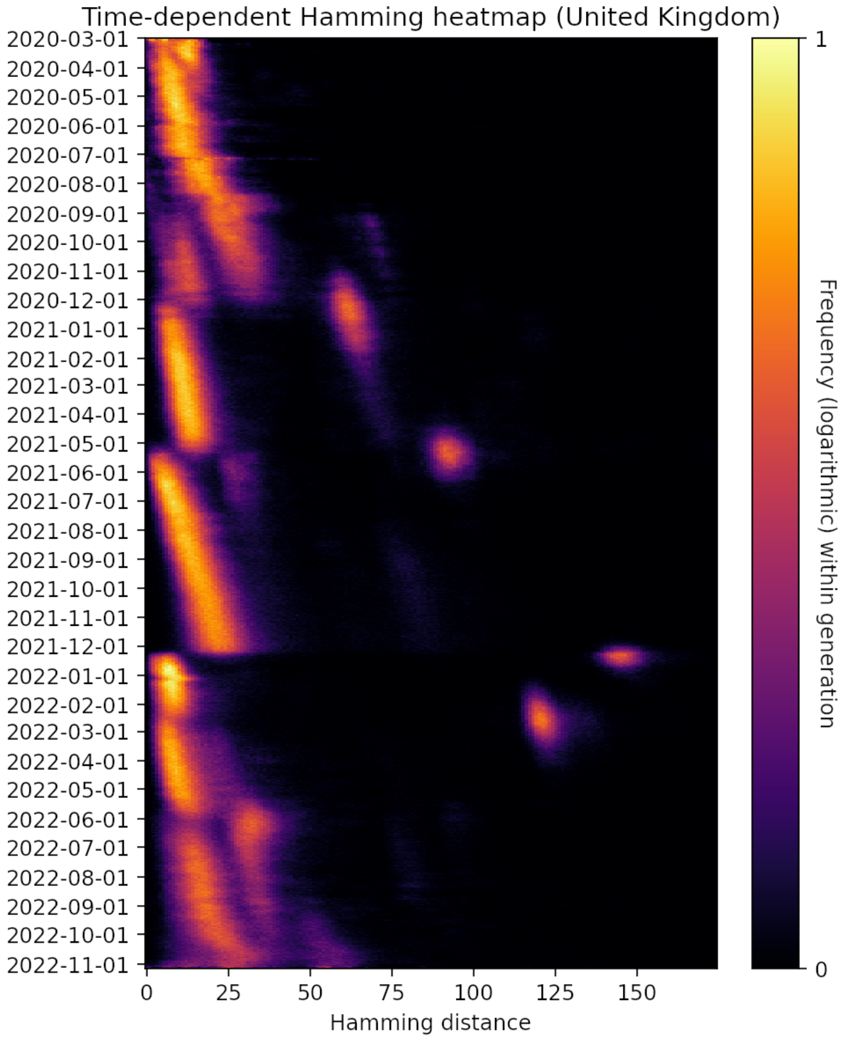
Time-dependent Hamming heatmap (United Kingdom)
2020-03-01
2020-04-01
2020-05-01
2020-06-01
2020-07-01
2020-08-01
2020-09-01
2020-10-01
2020-11-01
2020-12-01
2021-01-01
2021-02-01
2021-03-01
2021-04-01
2021-05-01
2021-06-01
2021-07-01
2021-08-01
2021-09-01
2021-10-01
2021-11-01
2021-12-01
2022-01-01
2022-02-01
2022-03-01
2022-04-01
2022-05-01
2022-06-01
2022-07-01
2022-08-01
2022-09-01
2022-10-01
2022-11-01
0
25
50
75
100
125
150
Hamming distance
1
0
Frequency (logarithmic) within generation

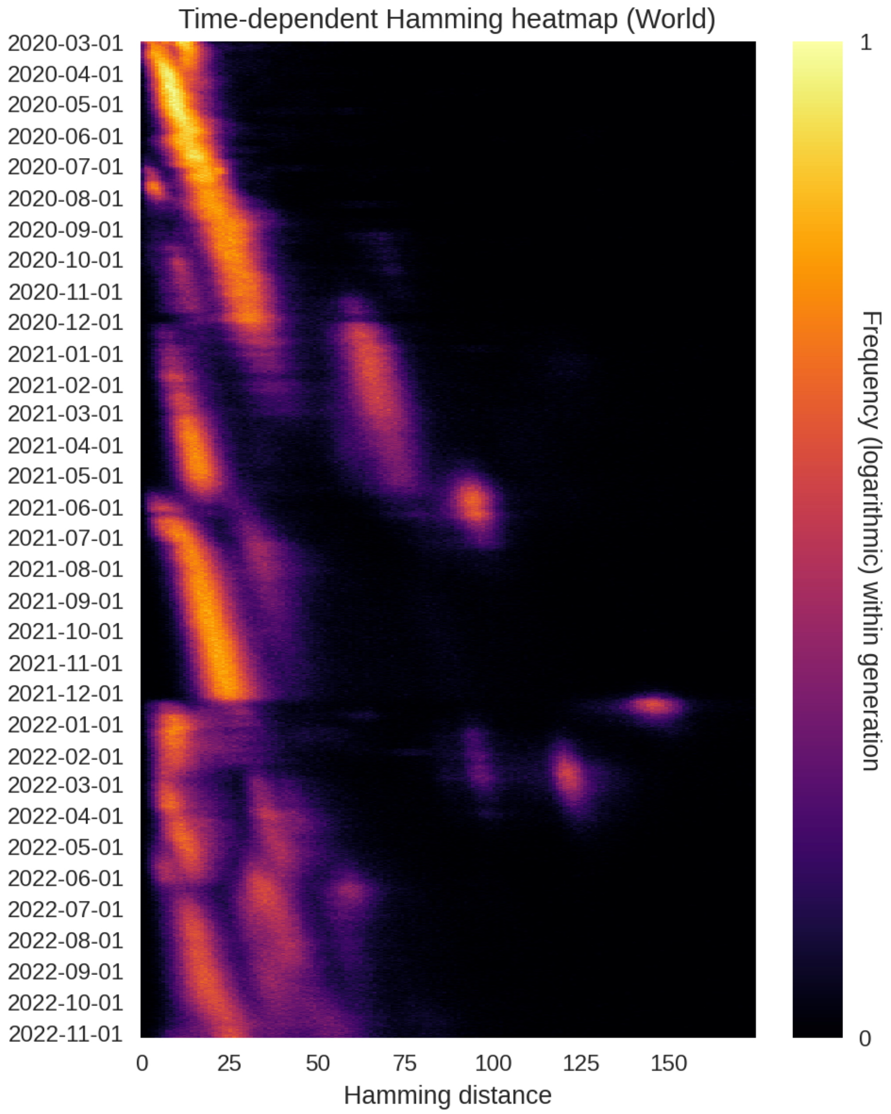

First of all, we see that the plot based on the entire World is somewhat "messier" than the UK one, but that the typical magnitudes of the Hamming distances involved are the same. This corresponds well to our observation in the manuscript of what spatial effects do to the simulated Hamming distributions (namely "smear out" the transitions). We have commented on this in the results section (and included the plot in supplementary figure S7).

We see that the Delta->Omicron BA.1 transition as well as the BA.1->BA.2 transition appeared as "clean" saltations, but that the picture has been a lot muddier since then.
In our understanding, this has to do with the pandemic essentially entering a new phase. While previous transitions happened in a background first of low population immunity (e.g. ancestral->alpha in the UK), then of high levels of vaccine-induced immunity (e.g. alpha->delta, delta->omicron BA.1), the more recent transitions have occurred in quite a different immunity landscape, characterised by high levels of vaccine coverage as well as immunity from prior infections. The immune landscape is thus very heterogeneous, with an appreciable fraction of the population having been exposed to e.g. BA.1, BA.2, BA.5 or a combination thereof.
If immunity has even a modest strain-specificity, this heterogeneous landscape would provide an evolutionary pressure for the concurrent (and rapid) appearance of new variants.

While a thorough treatment of this phenomenon is outside the scope of our paper, we have implemented a simple version of strain-specific immunity in our model to show how this affects the results.
In the simulations below, we assume that no mutations which increase inherent transmissibility occur. The only fitness-improving mutations are thus due to evasion of immunity. We do this to isolate the effects of (partially) strain-specific immunity.
We run the simulations within an (agent-based) SIRS scheme, i.e. with waning immunity.

First (left panel below), we assume 100% cross-immunity, i.e. that acquired immunity is completely strain-transcending/non-specific:

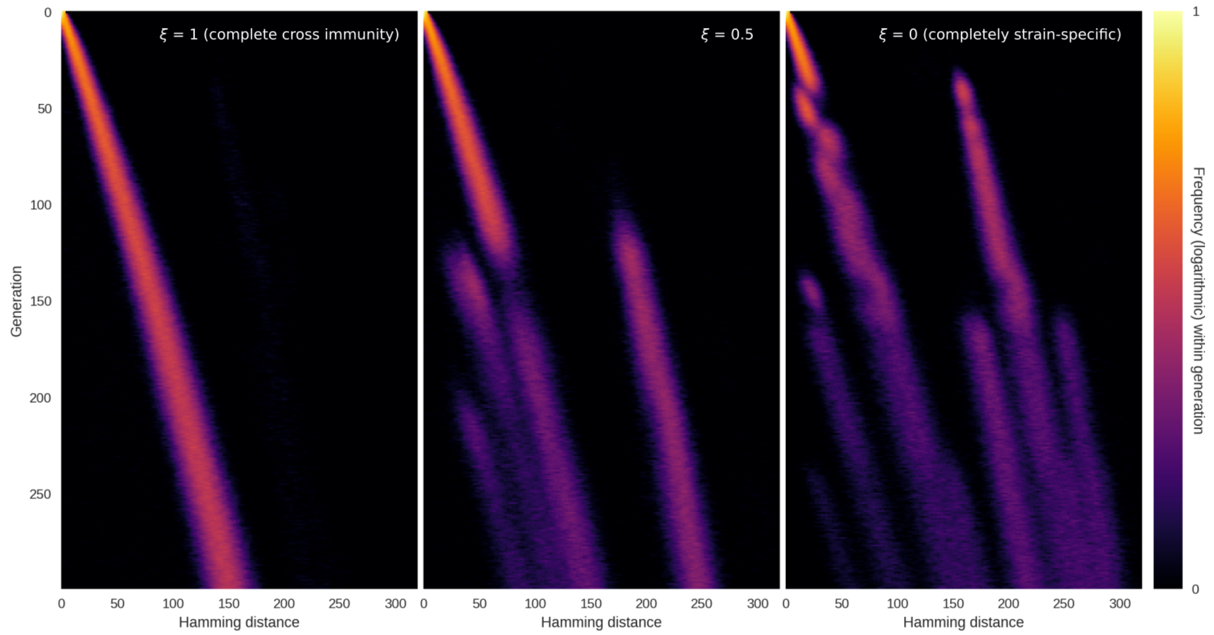

In this case, there is no pressure for new variants to emerge and the Hamming distance plot is dominated by the linearly increasing typical distance due to genetic drift (i.e. the molecular clock).
In the next simulation (middle panel), we assume only 50% cross-immunity, i.e. that immunity is somewhat strain-specific.
In this case, once immunity has built up in the population, there is a strong selective pressure for new variants to emerge. However, these new variants do not necessarily directly replace the previous variants, but may coexist with them due to a combination of strain-specific immunity and general waning. In the case of 0% cross immunity (i.e. complete strain-specificity), this tendency is even more pronounced (right panel above).
It should be noted that the magnitudes of the Hamming distances between the different strains depend on the particularities of how strain-transcending immunity is implemented (i.e. how different do strains have to be to evade immunity?).

We believe it pertinent to include a brief discussion of the current co-circulation situation in the manuscript and would like to thank the referee for raising this point. We have included a new appendix (Appendix 3) on this topic. We would like to defer a more thorough treatment of co-circulation and its origin in terms of the immunity landscape to future work, since this is a complex problem in its own right, separate from the saltational signature observed in the first several transitions during the pandemic.

Interestingly, if a new more intrinsically fit (i.e. more transmissible, higher R_0) variant arises in such an immunity landscape, it may or may not ‘refocus’ the Hamming distribution and replace previous variants, depending on the degree of cross immunity. See the plot below where a more transmissible variant arises at time t=250 with either no cross-immunity between strains (left panel) or 50% cross-immunity (right panel):

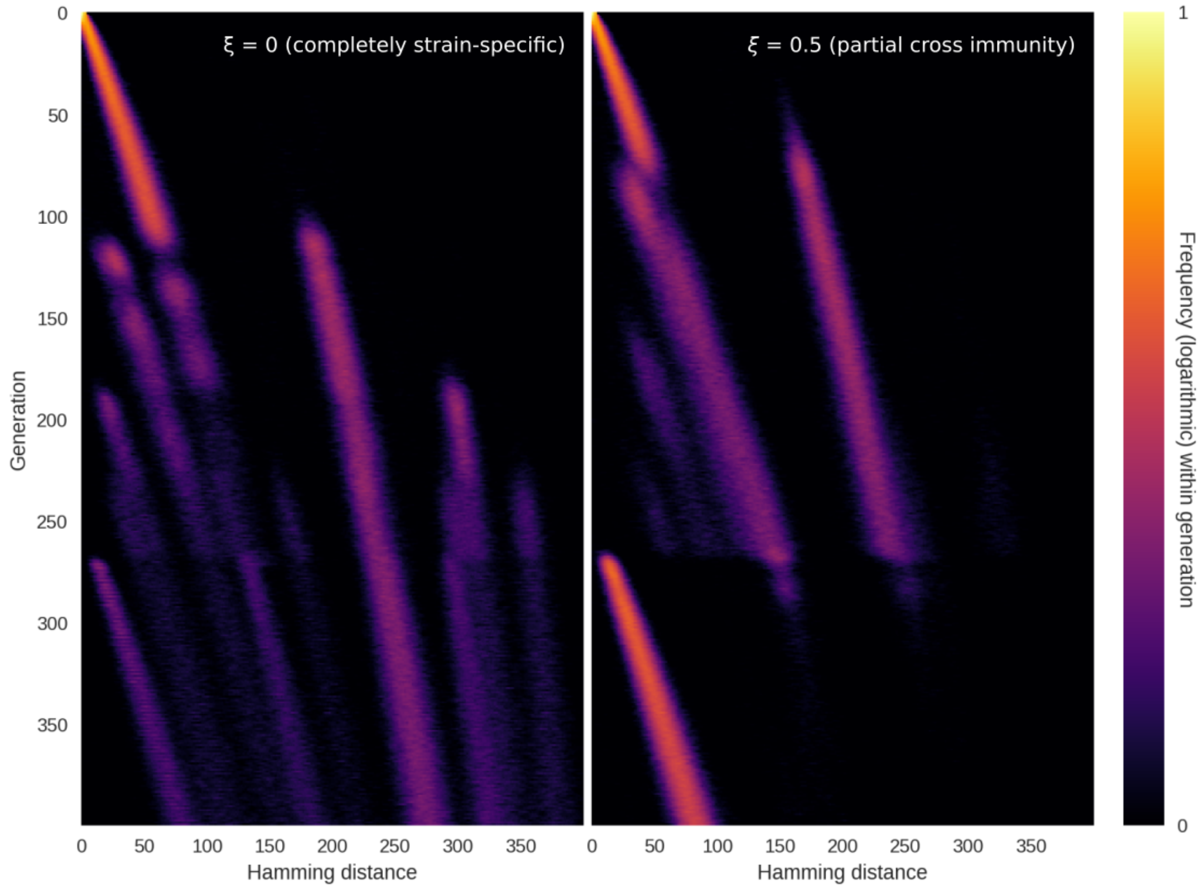

For a more thorough discussion of these simulations, we refer to Appendix 3.

Apart from the appendix on the possible origins of the post-BA5 variant co-circulation situation, we have updated the manuscript with the newest sequence data available to us.

(c) The interactions between groups were attempted to be addressed with the authors’ SEIR modeling, but those are smooth differential equations, and do not have the punctuated nature of the changes. Could the authors comment on what could happen in their model if they did not have saltational evolution, but rather the sudden appearance (in public) of people with different strains? Could that explain the punctuated rises and falls of genetic diversity?

Author response: Regarding the first point, our explanation of the implementation of SIRS dynamics was somewhat convoluted before. We had implemented SIRS dynamics by having a branching process (of variable generation size) and separately keeping track of the total number of susceptible and recovered individuals in aggregate.

We have since implemented a full agent-based (SIRS) version of the model, for a couple of reasons:

- It allows us to keep track of individuals regardless of their status as susceptible, infected or recovered
- It has allowed us to implement strain-specific immunity (with immune memory), which we have used for the new Appendix 3.

The agent-based implementation of SIRS dynamics is now described in the Methods section.
We still use a simpler, constant-prevalence branching process for our "main" figures (Figures 3, 4 and 5).

As for the question of the sudden appearance of people (in public) with different strains, we refer to Section 0 at the beginning of our reply.

Points a, b, and c above do not need a large change to the paper: just a sentence or so, say in the results or conclusions, to say whether this is an alternative effect that should be considered or if not, why not.
The introduction does an excellent job of saying that saltation might be the way to model the data, not that it must.

Author response: Thank you! This was indeed what we hoped to convey.

3) Figure 5 is puzzling somewhat: without saltation, the viruses in this model all die out. This is not what happens with most viruses; they persist in individuals and mutate, and come back to infect next season or next time someone has lowered immunity. The flu is here year after year, and certainly with a reproductive rate greater than one, and the authors do a good job of explaining how the model would be altered to model the flu well. Hepatitis is another one that persists, at high levels in some communities, without any saltation. Figure 4 is fine; what collapses after the genetic drift is the genetic diversity, not necessarily the viral population (which still presumably has a quasispecies distribution of some width). But Figure 5 seems to say that in its non-saltation version this model results in a non-functional virus. Perhaps the situation in Figure 5 can be clarified.

Author response: We admit that the original figure was perhaps a bit unclear: the plot gives the R_0 of the final strain *relative* to the original strain, i.e. $R_0^{final}/R_0^{initial}$, and the vertical axis starts at 1. So in the worst-case the fitness of the final strain is just unimproved relative to the ancestral. We agree that this was not particularly clearly indicated in the figure. To remedy this, we have now added a legend giving the above expression and have shifted the vertical axis to start at 0. The updated Figure 5 is included below for reference.
However, it should be said that Figure 5 only explores a situation where the fitness landscape is dominated by (sign) epistasis. This has allowed us to capture some of the dynamics for SARS-CoV-2, but we agree with the referee that this is probably not what determines season-to-season influenza evolution.

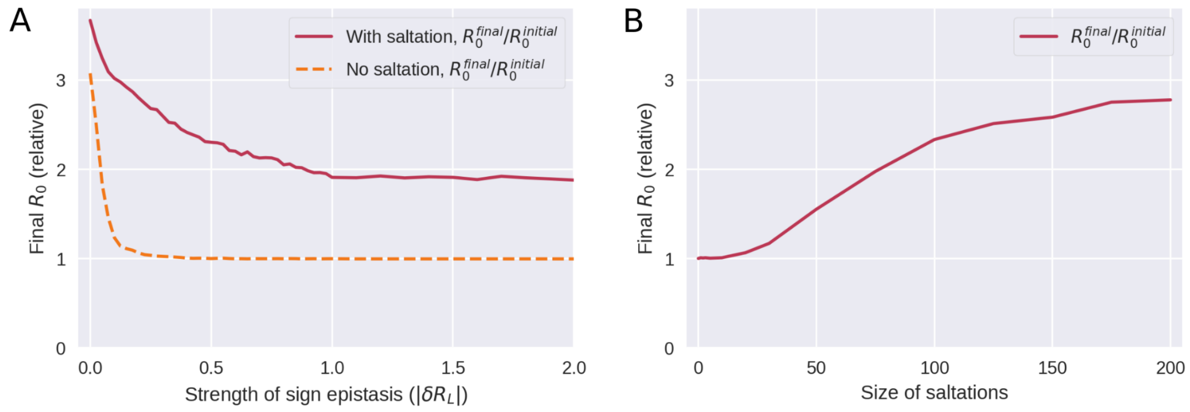

4) The branching part of the model could be explained with more clarity, in relation to Figure S3B. The size of the population N is not in Table I. The text describes the process fairly well, and Figure S3A is very good, but it is not clear how the images of Figure S3B relate to the description.

Author response: We have added some more annotations to Figure S3B to make it clearer (we agree that it was a bit opaque). Now we explicitly write that the two 'columns' are supposed to represent one generation and the next. We also specify that the arrows indicate transmission (with '*z* = *…*' giving the number of 'offspring'). We also reduced the number of individuals shown from each generation to three instead of five, to make the figure simpler (without any loss of information). See the new Fig S3 below.
We have also added both the size of the infected population (in the case of the constant-prevalence simulations) and the total population (in the case of the SIRS-type simulations) to the Table – thank you for making us aware of this omission.

A) Fitness landscape component

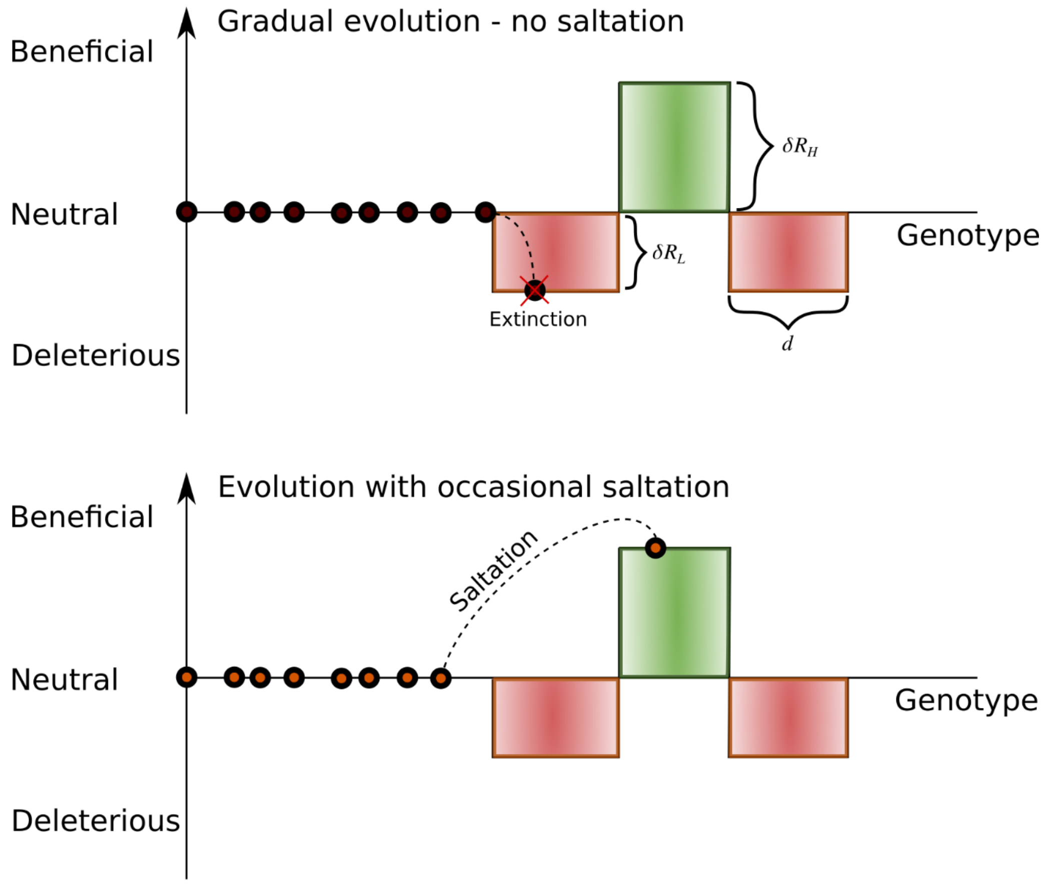

B) Branching model

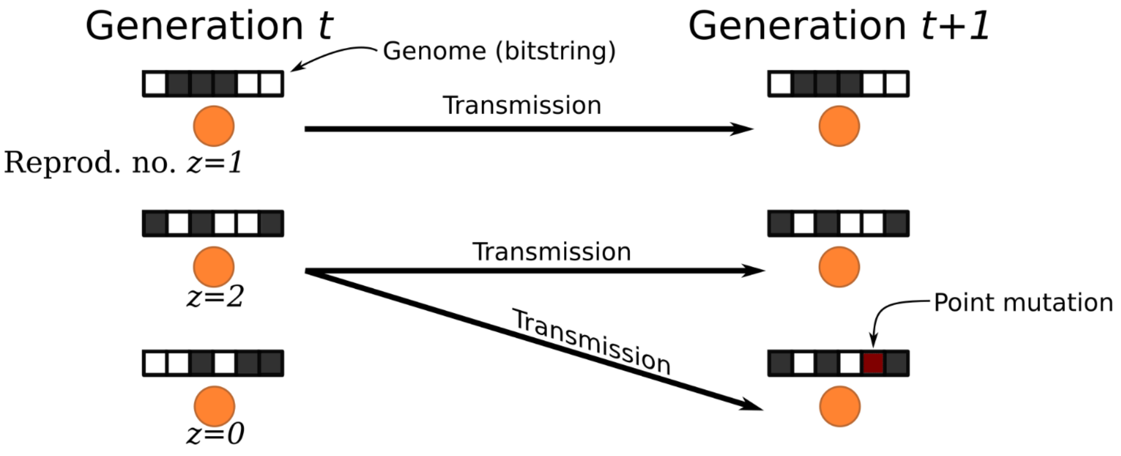

Supplement: Attachment — Submitted filename: ResponseToReviewers.pdf [file pcbi.1010896.s012.pdf]
